# Short-Term Effect of El Niño-Southern Oscillation on Pediatric Hand, Foot and Mouth Disease in Shenzhen, China

**DOI:** 10.1371/journal.pone.0065585

**Published:** 2013-07-23

**Authors:** Hualiang Lin, Hong Zou, Qinzhou Wang, Chunxiao Liu, Lingling Lang, Xuexin Hou, Zhenjun Li

**Affiliations:** 1 Guangdong Provincial Institute of Public Health, Guangzhou, China; 2 Center for Disease Control and Prevention of Guangdong Province, Guangzhou, China; 3 Baoan Chronic Diseases Prevent and Cure Hospital, Shenzhen, China; 4 Faculty of Medicine, the Chinese University of Hong Kong, Hong Kong SAR, China; 5 Shenzhen Entry-exit Inspection and Quarantine Bureau, Shenzhen, China; 6 State Key Laboratory for Infectious Disease Prevention and Control, National Institute for Communicable Disease Control and Prevention, China CDC, Beijing, China; The Australian National University, Australia

## Abstract

Hand, foot and mouth disease (HFMD) was an emerging viral infectious disease in recent years in Shenzhen. The underlying risk factors have not yet been systematically examined. This study analyzed the short-term effect of El Niño-Southern Oscillation on pediatric HFMD in Shenzhen, China. Daily count of HFMD among children aged below 15 years old, Southern Oscillation Index (SOI), and weather variables were collected to construct the time series. A distributed lag non-linear model was applied to investigate the effect of daily SOI on pediatric HFMD occurrence during 2008–2010. We observed an acute effect of SOI variation on HFMD occurrence. The extremely high SOI (SOI = 45, with 0 as reference) was associated with increased HFMD, with the relative risk (RR) being 1.66 (95% Confidence Interval [CI]: 1.34–2.04). Further analyses of the association between HFMD and daily mean temperature and relative humidity supported the correlation between pediatric HFMD and SOI. Meteorological factors might be important predictors of pediatric HFMD occurrence in Shenzhen.

## Introduction

Hand, foot and mouth disease (HFMD) is an emerging viral infection that usually affects infants and children. The main clinical presentation includes fever, mouth ulcers, and vesicles mainly on the hands, feet, and mouth [1]. In most cases, the disease is mild and self-limiting, but more severe clinical symptoms with neurological abnormalities such as meningitis, encephalitis, and polio-like paralysis may occur [2]. Currently, there is no vaccine or specific antiviral treatment available for HFMD. The most causes of HFMD are coxsackievirus A16 (CA16) and enterovirus 71 (EV71) [3], [4], among which, EV71 is more commonly linked with severe symptoms, including central nervous system disorders, and even death resulting from pulmonary edema in a small proportion of children, particularly those aged 5 years and younger [5], [6].

Outbreaks have been witnessed in the Asian Pacific regions in the past decade. In 2008, for instance, a large epidemic wave occurred in mainland China, Taiwan, Malaysia, Singapore, Hong Kong, etc [3]. The incidence of HFMD has been reported to exhibit seasonality in a number of different areas. For example, March-July peak was observed in Jiangsu Province, China [7]. Further, a bimodal seasonal pattern was reported in UK with peaks in summer and late autumn/early winter [8], while the highest incidence of HFMD in Taiwan was in summer [2]. Though the influence of climate on HFMD occurrence has long been debated [5], [9], the seasonality of HFMD incidence indicates that meteorological factors might have played a role in the epidemiology of HFMD. For example, in Hong Kong, it has been suggested that the changing epidemiology of HFMD (a new peak in winter) might be due to temperature increase in winter season [10]. In Japan, a time series study found that ambient temperature and relative humidity were significantly linked with increased HFMD occurrence [5]. Considering the seasonal pattern of HFMD, it was reasonable to hypothesize that short-term changes in weather variables may affect the transmission dynamic of HFMD.

As local weather conditions could be influenced by the global climate change [11], [12], we hypothesized that the dynamics of HFMD might be partly attributable to global warming, as the breeding, survival, transmission of the virus, population behavior as well as exposure patterns could be affected by climate change [9]. El Niño-Southern Oscillation (ENSO) is a systematic pattern of global climate variability [13]. Southern Oscillation Index (SOI), an indicator of ENSO activity, is defined as the normalized atmospheric pressure difference between Darwin in Australia and Tahiti in the South Pacific [14]. SOI has been reported to be related to a couple of infectious diseases, such as cholera, dengue fever and hemorrhagic fever with renal syndrome [15]–[17]. In China, negative SOI values are associated with El Niño conditions (dry and warm), and positive values link with La Niña conditions (wet and cool) [18]. So far, no study has been conducted to examine the effect of SOI on the transmission dynamic of HFMD.

The current study aimed to examine the relationship of day-to-day SOI variation with the occurrence of pediatric HFMD using surveillance data collected in Shenzhen, China, from 2008 to 2010. According to the surveillance data, this city had four-fold HFMD epidemic of the national average [19].

## Methods

### Setting

**Figure 1:**
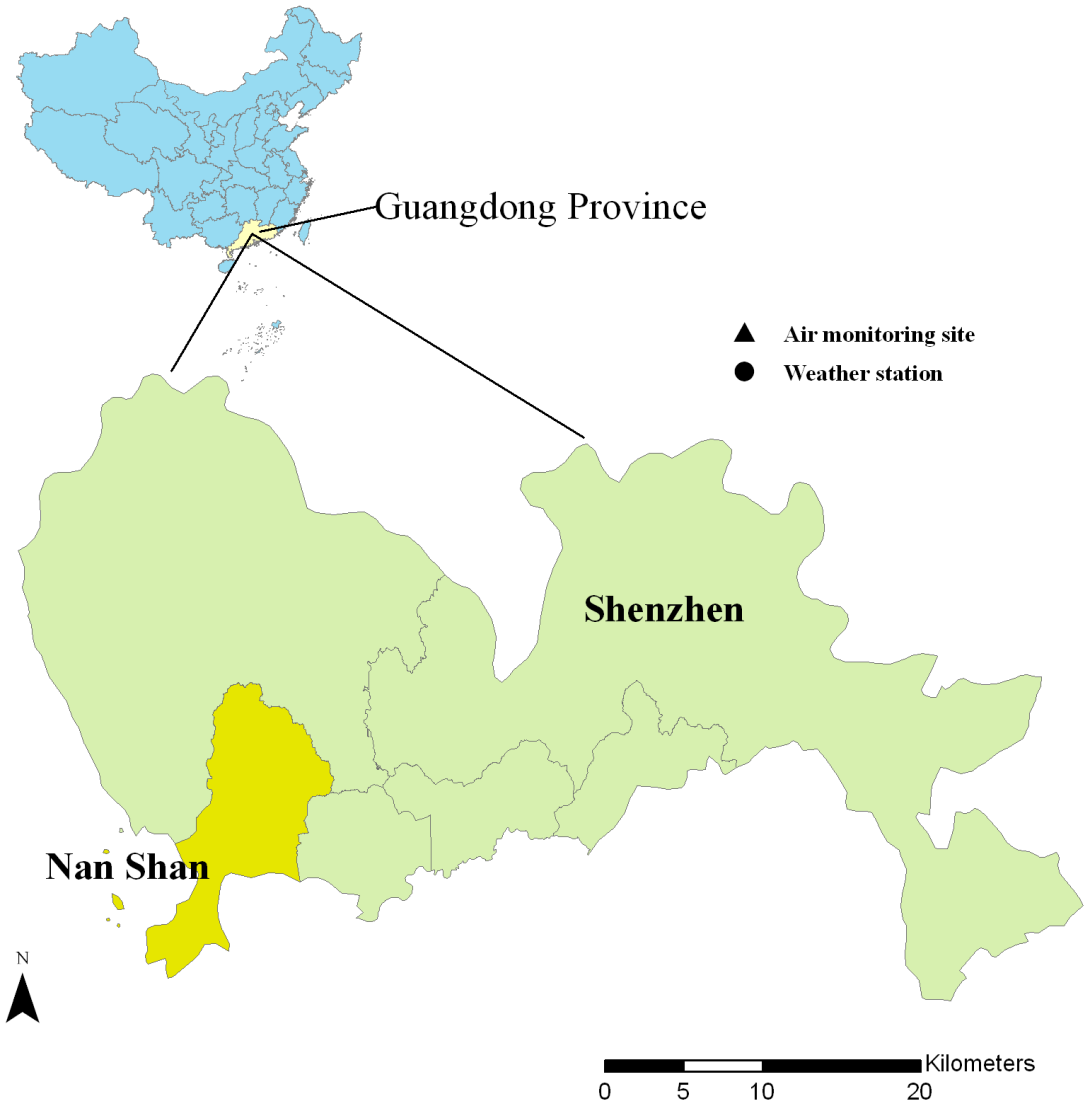
Geographical location of study area in China.

Shenzhen is a city in the south of Guangdong Province, adjacent to Hong Kong [20]. It has a typical monsoon-influenced climate with wet and hot summers and dry and cool to mild winters. Shenzhen has a population of 14 million. The residents of Nan Shan District were selected as the subjects for the present study, which has an area of 151 km^2^ and is home to 0.5 million residents. Nan Shan District was chosen for this analysis for two reasons. Firstly, there are weather and air pollution monitoring stations in this district. Secondly, the HFMD data in this district are of high quality. Figure 1 shows the geographical location of the study area in China.

### Data sources

Data on daily count of HFMD in children younger than 15 years old covering the period 2008–2010 were obtained from the local Center for Disease Control and Prevention. HFMD was diagnosed by clinical symptoms, which included vesicular lesions on hands, feet, mouth (which were often ulcerated), and, frequently, buttocks, in accordance with the National Guideline on Diagnosis and Treatment of Hand Foot Mouth Disease (Chinese Ministry of Health). According to China's notifiable infectious disease regulation, all HFMD cases were required to be reported to the infectious disease surveillance system via the web-based surveillance system with standardized format, including the information of name, sex, age, address, date of symptom onset, etc..

Daily SOI data were publicly accessible from the Australian Bureau of Meteorology (http://www.longpaddock.qld.gov.au/). Meteorological data including daily mean temperature and relative humidity were obtained from National Weather Data Sharing System (http://cdc.cma.gov.cn/home.do), which was publicly accessible. As some studies have suggested that particulate air pollution might be related with the transmission of the virus, we therefore collected the daily PM_10_ (particulate matter with aerodynamic diameter less than 10 micrometers) concentration data from the local air monitoring department. The geographical distribution was shown in Figure 1.

### Statistical analysis

As the daily number of HFMD generally followed a Poisson distribution, a time series approach [21] that has been generalized to investigate the distributed lag non-linear association was performed to examine the effect of daily SOI on daily HFMD and its associated lag structure. Briefly, we used quasi-likelihood Poisson regression in a generalized linear model to fit the natural logarithm of daily counts of HFMD cases as functions of the predictor variables. The approach accounted for the over-dispersed Poisson data using the assumption that the total variance was proportional to the total number, with the over-dispersion constant estimated through quasi-likelihood.

We made use of the distributed lag non-linear model to examine simultaneously non-linear and delayed structure in the association between daily pediatric HFMD and SOI. The methodology was based on the definition of a “cross-basis” function, which allowed the non-linear effect of daily SOI at each lag and the nonlinear effects across lag periods to be estimated [22]. We used a “primary” model to construct the model and we did sensitivity analyses to investigate the robustness of the effect estimates. The “primary” model had a natural cubic spline with 5 df in the lag space and a cubic b-spline with 2 df in the SOI space. We used lags up to 30 days in order to capture the overall effects according to a previous study [23]. Potential confounding factors were controlled for in the model, which included an indicator for day of week (DOW), an indicator of public holidays (PH), natural spline for time (6 df/year) in order to control for the seasonal effect and long-term trend, a smooth function of mean temperature (6 df), a smooth function of relative humidity (3 df), and smooth function for particulate air pollution (3 df). For all of the smooth functions, we used a natural spline basis. We reported the effects of SOI on HFMD along certain lags with 0 as the reference. The model used for the analysis could be specified:
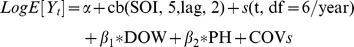
where E(Yt) denoted the expected daily HFMD count on day t, cb meant the “cross-basis” function, s(•) indicated a smooth function based on natural splines for nonlinear variables, β was regression coefficient, and COVs were the potential confounding factors.

### Sensitivity analysis

As the risk estimates usually varied with the model specifications in time-series analysis [24], [25], we performed additional sensitivity analyses to test the robustness of our results: use of alternative degrees of freedom (5, 7 df/year) for temporal adjustment and use of alternative degrees of freedom for meteorological variables and particulate air pollution.

All statistical analyses were two-sided and values of P<0.05 were considered statistically significant. The dlnm package [21] in R software Version 2.14.1 (R Development Core Team, 2012) was used to fit all models and estimate the exact standard errors of regression coefficients.

## Results

**Figure 2:**
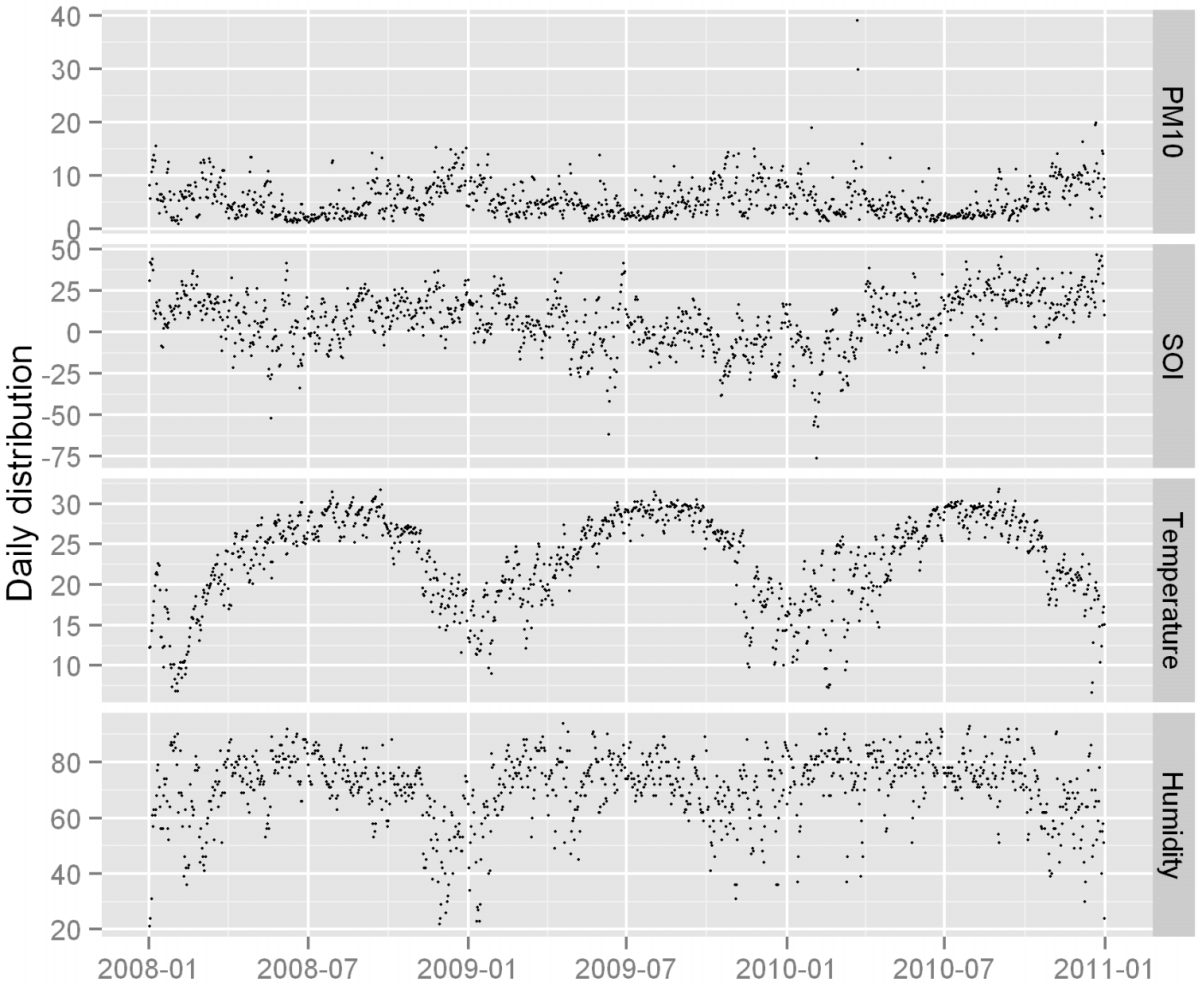
The time series of daily weather condition, SOI and PM_10_ in Nan Shan, Shenzhen, 2008–2010.

Between 1 January 2008 and 31 December 2010, there were a total of 3911 pediatric HFMD cases reported in Nan Shan District, Shenzhen. There were more male cases with a male-to-female sex ratio of 1.6∶1 (2394∶1517). The descriptive summary for weather conditions and particulate matter air pollution was shown in Table 1. There was an average of 3.6 daily pediatric HFMD cases over the study period. Daily PM_10_ concentration was 54.6 ug/m^3^, the mean level of daily SOI, mean temperature and relative humidity were 6.5, 23.0°C and 70.8%, respectively. Figure 2 depicted the time series of weather conditions and PM_10_ concentration in the study area. There were seasonal patterns in these factors.

**Table 1:** Summary statistics of daily weather condition, SOI, PM_10_ and pediatric HFMD count in Nan Shan District, Shenzhen, 2008–2010.

Variables	Min	Mean	Max	SD
Daily SOI	−76.0	6.5	46.6	16.7
Mean temperature (°C)	6.7	23.0	31.8	5.6
Relative humidity (%)	21.0	70.8	94.0	12.9
Daily HFMD count	0.0	3.6	77.0	6.5
PM_10_ (ug/m^3^)	8.9	54.6	391.4	34.9

SD: standard deviation.

**Figure 3:**
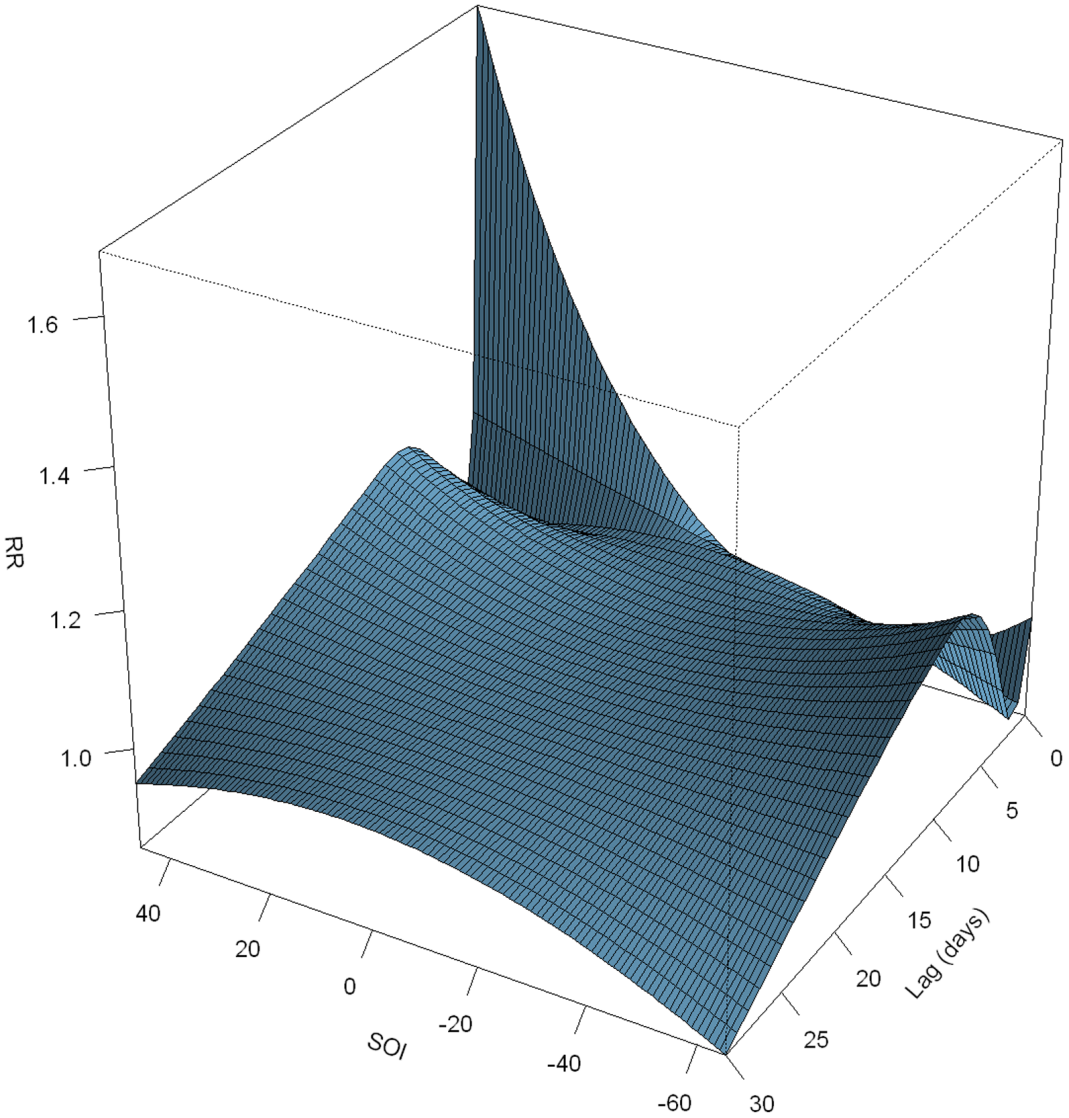
Three-dimension plot of relative risk along daily SOI and lag days, with reference at 0 SOI.

An overall picture of the effect of daily SOI on pediatric HFMD was illustrated in Figure 3, showing a three-dimensional plot of the relative risk (RR) along daily SOI and lags with 0 as the reference. The plot showed a strong and immediate effect of high SOI on HFMD occurrence, the effect declined sharply and lasted for about 3 days. Inspection of the figure at longer lag days suggested no harvesting effect. We further calculated the relative risk for the high SOI (with 45 as example) at the current day to lag 3 days, and found that only high SOI level on the current day was significantly associated with increased HFMD occurrence. The relative risk for high SOI (45) was 1.66 (95% CI: 1.34–2.04).

**Figure 4:**
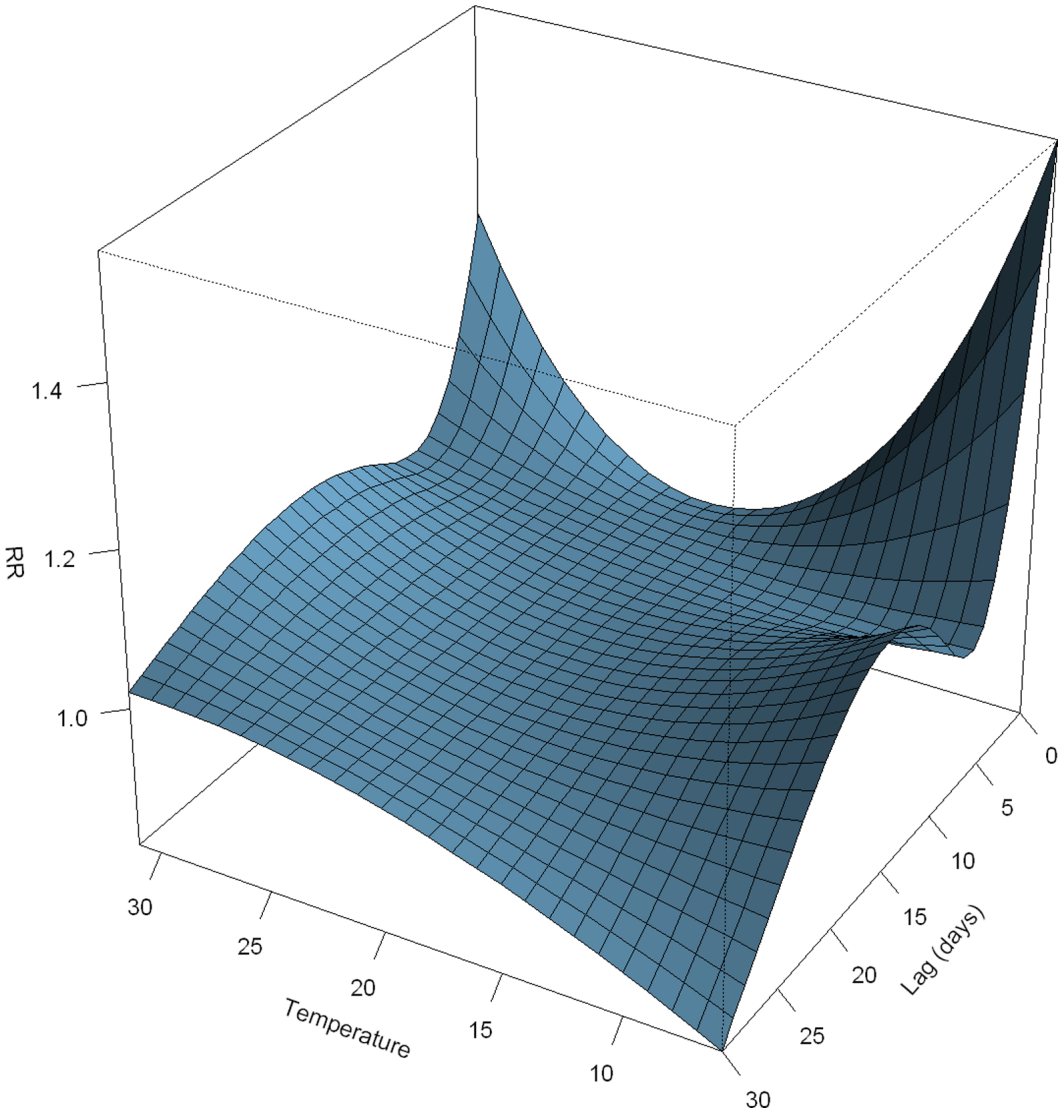
Three-dimension plot of relative risk along daily temperature and lag days, with reference at 23°C.

As a positive SOI indicated wet and cold weather condition in the study region, we further examined the association of daily HFMD with both daily mean temperature and relative humidity to check whether the observed effect of SOI was supported by that of local weather variables. Figure 4 showed the overall association between daily mean temperature and HFMD. A U-shape relationship was observed on the current day, with both high and low temperatures having an acute effect on HFMD risk, and low temperature resulting in the largest relative risk (e.g., the RR for 10°C was 1.46 (95% CI: 1.10–1.90) with the daily mean temperature (23°C) as a reference).

**Figure 5:**
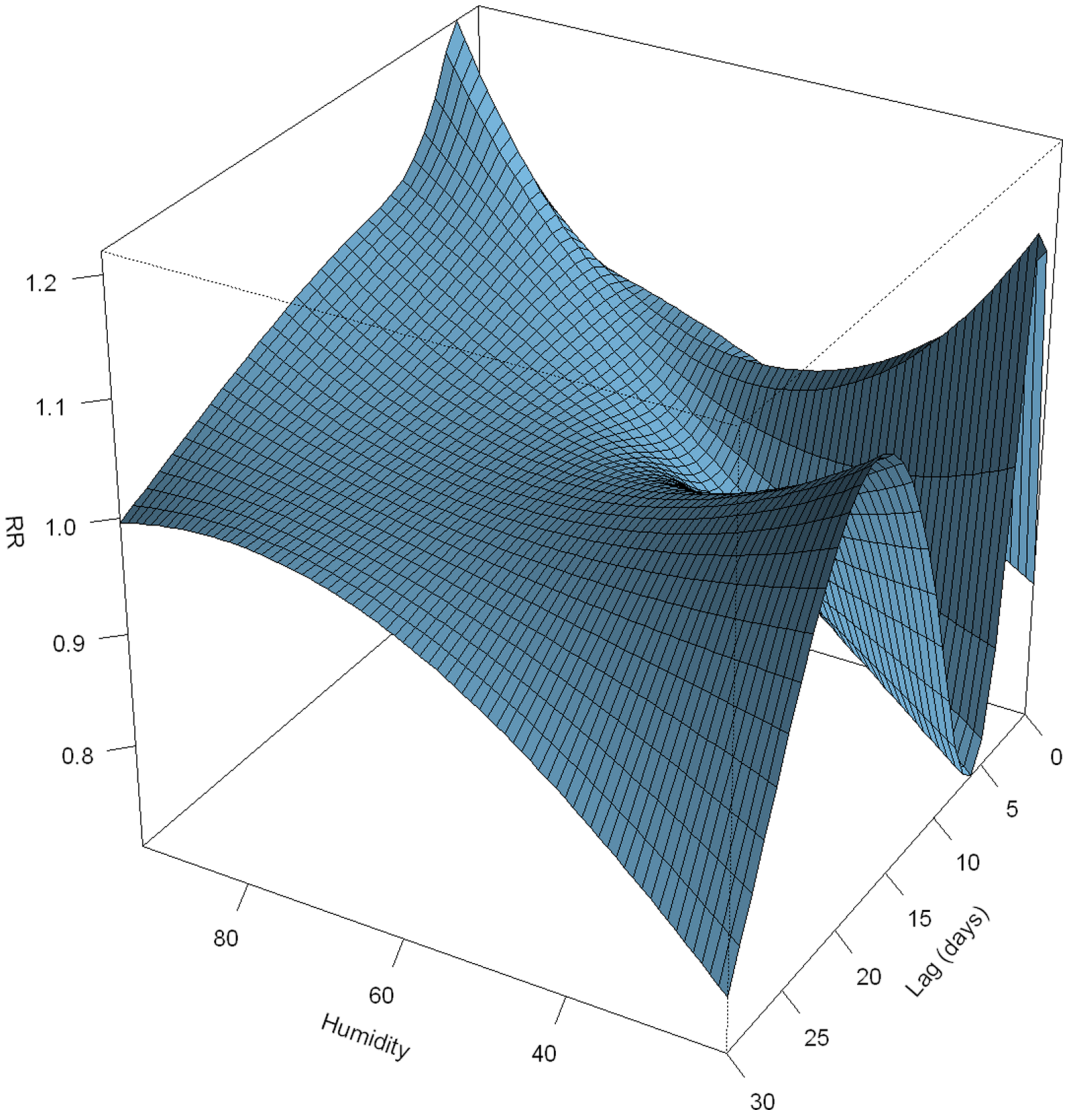
Three-dimension plot of relative risk along daily relative humidity and lag days, with reference at 71%.

The relationship between daily relative humidity and pediatric HFMD was shown in Figure 5. We found that high relative humidity (wet weather condition) at lag 1 day was associated with increased HFMD occurrence (for example RR for relative humidity being 90% was 1.13 (95% CI: 1.05–1.21) with 70.8% as a reference).

In the sensitivity analyses, when the degrees of freedom for time were varied between 5 and 7 to control for seasonality and long-term trends, similar results were obtained. Furthermore, the estimated effect of SOI did not change substantially when the degrees of freedom for meteorological factors and particulate air pollution were varied between 4 and 7.

## Discussion

The mounting evidence for rapid global climate change has highlighted the necessity to investigate the relationship between weather variability and infectious diseases [26], [27]. Our results showed that the Southern Oscillation Index was related to day-to-day variation of hand, foot and mouth disease in Shenzhen and suggested that meteorological factors might play an important role in the transmission dynamic of HFMD in the study region. According to our findings, the daily count of HFMD in Shenzhen tended to reach its highest level when the SOI value was highest. The observed association between HFMD and both mean temperature and relative humidity supported the association between HFMD and SOI, suggesting that the findings were robust.

Due to the complex interactions involved in the weather indicators, it was difficult to directly explain the observed association between SOI and HFMD occurrence. However, some climatic and epidemiologic behaviors related to HFMD could be useful in drafting a possible hypothesis about the pathways by which the SOI could affect the day-to-day variation of HFMD. It was possible that when SOI value was positive, the weather conditions (wet and cold) in the study area were favorable for the survival and transmission of the HFMD virus in the environment, thereby increasing the possibility of transmission to the children. Positive SOI was usually associated with humid weather in the study region. In the study area, more than 67% of pediatric HFMD cases have been reported in April-July, during which the weather was windy and rainy. The result of the study demonstrated that relative humidity was also related to an increase in HFMD. These results were consistent with findings in different regions of the world [10], [28].

HFMD was mainly transmitted through the faecal-oral route and respiratory droplets. There have been studies suggesting that particulate matter air pollution might play a role in the air-borne virus transmission, particularly influenza [29], [30]. It was possible that during the humid and rainy days, the virus could be easily attached to the small particles in the air and then facilitated the disease transmission [31]. However, this detailed mechanism needed to be confirmed in future studies.

The findings of this study were especially interesting given the expected increasing influence of global climate change on the local weather conditions [11]. The present study provided the first evidence of the relationship between the SOI and hand, foot and mouth disease occurrence. Better understanding of this complex relationship, particularly of the impact of SOI on the population behavior and susceptibility and transmission of the disease pathogen, could provide additional tools to predict its epidemic risk [32], [33]. The ability to forecast HFMD on the basis of day-to-day SOI variation could permit preventive improvements on public health infrastructure, including access to health care resources, scientific knowledge, active disease surveillance, and designing more specific control measures to mitigate the disease transmission risk.

Our study had two major strengths. Firstly, this study investigated the impacts of Southern Oscillation Index on hand, foot and mouth disease in Shenzhen using an advanced statistical approach (dlnm) [21], [24], [34]. The distributed lag non-linear approach could flexibly examine the possible non-linear relationship and lag effects simultaneously [21]. Although this model was relatively complex and had many parameter specifications, our sensitivity analyses suggested that the results of our study were insensitive to the model specification. Secondly, consistent findings of local weather variables with that of SOI indicated that our results might be a possible mirror of the real picture of the relationship between SOI and pediatric HFMD occurrence.

On the other hand, a few limitations should be considered when interpreting findings from this study. Firstly, our study was ecological in study design nature which did not allow us to explore individual-level association and limited our capacity for causal inference [25]. Secondly, our analysis was preliminary and exploratory, we could not exclude the possibility that unmeasured confounding factors, that were associated with both SOI and HFMD occurrence, might have affected our finding.

In conclusion, our study suggests that positive SOI may have harmful effects on pediatric HFMD occurrence in Shenzhen. Meteorological variables might be important predictors of pediatric HFMD occurrence in the study area.
